# Time-dependent variation of ionized calcium in serum samples

**DOI:** 10.11613/BM.2019.030708

**Published:** 2019-10-15

**Authors:** Antonija Perović, Marina Njire Bratičević

**Affiliations:** Department of Laboratory Diagnostics, Dubrovnik General Hospital, Dubrovnik, Croatia

**Keywords:** ionized calcium, serum, heparin, specimen handling, pre-analytical phase

## Abstract

**Introduction:**

The aim of this study was to compare ionized calcium (iCa) concentrations in arterial heparinized blood and venous serum and to investigate time-dependent variation of iCa in serum samples centrifuged and analysed at different times.

**Materials and methods:**

Ionized calcium was measured (N = 25) in arterial blood within 20 min after puncture, and in serum within 10 min after centrifugation conducted 30 min after sampling. Effect of time between sampling and centrifugation was examined in three tubes (N = 30) centrifuged 15, 30 and 60 min after sampling, and analysed within 10 min. Effect of time between centrifugation and analysis was investigated in three tubes (N = 31) centrifuged 30 min after sampling and analysed: 0-10, 30-40 and 90-100 min after centrifugation. Ionized calcium was measured on the Siemens RapidLab 348EX analysers. Statistical significance was tested using Wilcoxon test and ANOVA analysis. Clinical significance was judged against reference change values (RCV).

**Results:**

No statistically significant difference was found between iCa in arterial blood and serum (P = 0.274). A statistically significant decrease was found: in tubes centrifuged 60 and 15 min after sampling versus 30 min (P = 0.005, P = 0.003); and in tubes analysed 30-40 and 90-100 min after centrifugation versus 0-10 min (P = 0.021, P = 0.027). Clinically significant changes were observed: 60 versus 30 min (centrifugation) and 90-100 versus 0-10 and 30-40 min (analysis).

**Conclusions:**

Timely analysed arterial blood and serum samples can be used interchangeably. To avoid clinically significant variations, serum tubes should be centrifuged within 30 min after sampling, and analysis should be performed within 30 min after centrifugation.

## Introduction

It is well recognized that the free or ionized calcium component (iCa) is a better indicator of the calcium physiological status in blood than total calcium (*1*). Measurement of iCa plays an important role particularly among patients with primary hyperparathyroidism, late stages of chronic kidney disease (CKD), in patients receiving transfusions with citrated blood, in critically ill patients, and hypercalcemia of malignancy (*2*-*4*). Unfortunately, method availability, analytical performance, standardization, practicality of sample handling and lack of automated analysis often limit determination of iCa in a clinical laboratory (*3*).

After sampling, iCa concentration is mainly influenced by pH and various ligands such as bicarbonate, lactate, phosphate and anticoagulants (*5*). The pH increase leads to a higher binding of iCa to proteins and other anions, decreasing iCa concentration (approximately 0.04 mmol/L *per* 0.1 pH units) (*1*, *6*). The change in iCa concentration in the sample is a dynamic processes influenced by factors such as: lactate creation due to continued cell metabolism, loss of carbon dioxide during clotting, initial pH value, protein and calcium concentration in the sample (*6*-*9*). Also, it is well known that heparin may affect iCa concentration and may underestimate iCa concentration in whole blood or plasma compared to serum concentrations (*5*, *7*, *10*, *11*). Calcium binding to heparin can be minimized either by using a low heparin concentration or by using heparin titrated with calcium or balanced with other ions (*5*, *7*, *12*). Therefore, it is important that iCa determination in whole blood is performed on samples collected in syringes or capillaries anticoagulated with the modified heparin preparations designed to minimize the effect of iCa binding to heparin (*7*).

According to the Clinical and Laboratory Standards Institute (CLSI), recommended samples for iCa measurement are heparinized whole blood and anaerobically collected serum (*7*). An advantage of heparinized whole blood is the sample availability for analysis immediately after collection, and thus the reduced effect of cell metabolism on iCa due to rapid analysis (*5*, *7*). On the other hand, the disadvantage is the sample instability and inability to delay the analysis. Furthermore, undetected haemolysis or higher heparin concentration due to incomplete filling of the blood-collection device can artificially decrease the measured iCa concentration (*7*, *13*). The advantages of using serum in relation to whole blood are the sample stability, easy detection of haemolysis and the sample contains no anticoagulants that may bind calcium ions, while the disadvantages are related to delayed analysis and continuation of cellular metabolism during clotting and centrifugation (*7*).

Therefore, the aim of this study was to compare the concentrations of iCa in timely analysed samples of arterial whole blood and venous serum. Since many blood samples are collected outside the laboratory where the tests are performed and transported in non-centrifuged or centrifuged serum tubes, the second aim of this study was to examine the effect of time between sampling and centrifugation and between centrifugation and analysis on iCa concentration in the serum sample.

## Materials and methods

### Study design

The study was conducted in the Department of Laboratory Diagnostics, Dubrovnik General Hospital, Dubrovnik, Croatia from March to May 2015, and from October to December 2018. The study protocol was explained to all participants and they all signed informed consent. The Institutional Ethical Committee approved the study which was carried out in accordance with the Helsinki declaration. The criteria for exclusion from the study were the presence of haemolysis in samples that were checked visually according to haemolysis scale, and the incompletely filled syringes or tubes up to the nominal volume.

To compare iCa concentration in arterial blood and venous serum, blood samples were collected from 25 subjects (10 women and 15 men; median age: 45 years; range: 27 to 61 years). Venous samples were collected directly into plastic vacuum tubes (5 mL) with clot activator and gel-separator (Vacuette, Greiner Bio-One GmbH, Kremsmünster, Austria). All tubes were mixed according to the manufacturer’s recommendation, left in an upright position for 30 min at room temperature, and then centrifuged at 2200xg for 10 min (*14*). The serum of each blood sample was analysed within 10 min after centrifugation. Immediately after venipuncture, arterial puncture from the radial artery was performed using a 3 mL plastic RAPIDLyte syringe with ~ 70 IU of balanced heparin (Siemens Healthcare Diagnostics Inc., Tarrytown, USA). The iCa concentration in arterial samples was determined within 20 min after arterial puncture. After analysis, an aliquot of the arterial blood was centrifuged to check the presence of haemolysis.

The second phase of this study was to investigate the effect of time between sampling and centrifugation on iCa concentration in the serum samples obtained from 30 subjects (21 women and 9 men; median age: 35 years; range: 25 to 47 years). From each subject, three tubes of venous blood were taken. To minimize the effect of cellular metabolism during clotting, the first tube (Tube 1) was centrifuged 15 min after sampling (time when the beginning of the blood clotting is commonly observed). The second tube (Tube 2) was centrifuged according to the manufacturer’s recommendation, *i.e.* 30 min after sampling (*14*), while the third tube (Tube 3) centrifuged 60 min after sampling - time at which the serum should be separated from the cells according to the recommendation of the International Federation for Clinical Chemistry (IFCC) (*5*). After centrifugation, in all tubes, iCa concentration was determined within 10 min.

To determine the time required for delivery of centrifuged serum tubes at room temperature (from other laboratories in our town), the third phase of the study examined the effect of time between centrifugation and analysis on iCa concentration. Three venous blood samples were obtained from each of 31 subjects (22 women and 9 men; median age: 37 years; range: 25 to 50 years). All tubes were centrifuged 30 min after blood sampling, according to the manufacturer’s recommendations (*14*). The tubes were analysed at the following times: within 10 min (Tube A), between 30 and 40 min (Tube B) and between 90 and 100 min after centrifugation (Tube C).

In the period between sampling and analysis, all arterial and venous blood samples were left at room temperature (22-25 °C) and were not opened at any time until analysis. Concentrations of iCa were measured by potentiometric method on the RAPIDLab 348 analysers (Siemens Healthcare Diagnostics Inc., Tarrytown, USA). Prior to the analysis, arterial samples were visually inspected for the presence of air bubbles or visible clots and were gently rotated for a minimum of one min. After the serum tubes were opened, the serum was carefully aspirated from the serum layer above the separator gel which was not exposed to air. In each sample iCa concentration was determined on two analysers, and the mean value of the two measurements was used for statistical analysis.

### Statistical analysis

Normality of data distribution was accessed using the Shapiro-Wilk test. A statistically significant difference between iCa concentrations in arterial blood and serum samples was tested using non-parametric Wilcoxon signed-rank test, and the results were presented as the median and interquartile range (IQR). A statistically significant difference between iCa concentrations in serum tubes that were centrifuged and analysed at different times was tested using the ANOVA analysis for repeated measures with Bonferroni post-hoc analysis, and the results were expressed as mean ± standard deviation. Comparison of iCa concentrations was evaluated using Bland-Altman plot and Passing-Bablok regression analysis including the Cusum test for linearity. Level of significance for all statistical comparisons was set as P < 0.05. Statistical analyses were performed using MedCalc statistical software version 16.4.3 (MedCalc, Ostend, Belgium).

### Clinically significant change

An analytically significant difference between iCa concentration in the examined samples was assessed according to the criterion from the Croatian Centre for Quality Assessment in Laboratory Medicine (CROQALM), which is based on the minimum total error (4%) calculated from the biological variation (*15*, *16*). A clinically significant change was judged against calculated reference change value (RCV). Calculated RCV was 6.25%, according to the equation: RCV = 2^1/2^ × Z × (CV_A_^2^ + CV_I_^2^)^1/2^ where Z is 1.96 for the desired probability of P < 0.05 and CV_I_ is the within-subject biological variation from the database on biological variation (*16*). CV_A_ is the analytical coefficient of variation calculated from our laboratory’s daily internal quality control results during the study period from March to May 2015, and from October to December 2018, for both analysers (1.49% and 1.47%, mean value 1.48%). Internal quality control was performed twice a day using the manufacturer’s controls RAPIDQC Plus Level 1, 2 and 3, (Siemens Healthcare Diagnostics Inc., Tarrytown, USA). The concentrations of the internal quality control materials were 1.62 to 1.66 mmol/L for level 1, 1.20 to 1.26 mmol/L for level 2, and 0.74 to 0.82 mmol/L for level 3. In the study period, comparison of two RAPIDLab 348 analyzers, which was performed twice a day using patient samples, was less than 2%. The bias between the two analysers and CV_A_ were judged against quality specifications for desirable total error (2%) from the database on biological variation (*16*).

## Results

**Figure 1:**
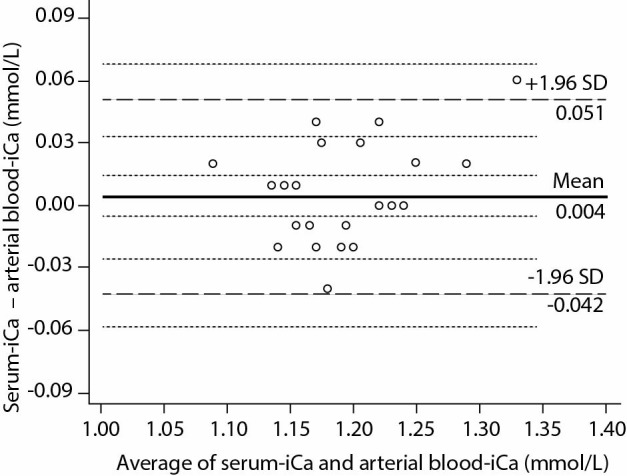
Bland-Altman plot for iCa concentrations between samples of arterial whole blood and venous serum. Solid line - mean difference (absolute value). Dashed lines - limits of agreement. Dotted lines - 95% confidence interval limits for mean difference and agreement limits. iCa - ionized calcium.

Comparison of iCa concentrations between arterial blood and serum samples is presented in Figure 1 and Table 1. No statistically significant difference was found between iCa concentrations in arterial blood and serum (P = 0.274), while statistically significant difference for pH values was observed (P < 0.001) (Table 1). ANOVA analysis for repeated measures showed a statistically significant difference for iCa and pH values between the serum tubes that were centrifuged and analysed at different times (Table 2). In relation to iCa concentrations obtained from the tubes that were centrifuged according to the manufacturer’s recommendation, a statistically significant decrease was found in tubes that were centrifuged 15 min after sampling (Tube 1 *vs.* Tube 2; P = 0.003) and in tubes that were centrifuged 60 min after sampling (Tube 3 *vs.* Tube 2; P = 0.005). A statistically significant decrease for iCa concentrations was also found in Tubes B (analysed 30-40 min after centrifugation), and in Tubes C (analysed 90-100 min after centrifugation) in relation to the Tubes A (analysed within 10 min after centrifugation) (P = 0.021, P = 0.027). Data analysis using Passing and Bablok regression (Table 3) showed the presence of constant and proportional differences between Tubes A and C. The presence of constant difference was also found between Tubes B and C.

**Table 1:** Comparison of ionized calcium and pH in arterial whole blood and venous serum

**Parameter, unit**	**Arterial blood**	**Serum**	**P**
iCa, mmol/L	1.20 (1.16-1.21)	1.19 (1.16-1.22)	0.274
pH	7.419 (7.409-7.426)	7.373 (7.362-7.384)	< 0.001
Data presented as median and interquartile range. The difference was tested using Wilcoxon test. P < 0.05 was considered statistically significant. iCa - ionized calcium.			

**Table 2:** Effect of time between sampling and centrifugation and between centrifugation and analysis on ionized calcium and pH in serum samples

	**Time from sampling to centrifugation (N = 30)**			**P**		
**Parameter, unit**	**15 min** **(Tube 1)**	**30 min** **(Tube 2)**	**60 min** **(Tube 3)**	**Tube 2 *vs.* Tube 1**	**Tube 3 *vs.* Tube 1**	**Tube 3 *vs.* Tube 2**
iCa, mmol/L	1.30 ± 0.04	1.31 ± 0.05	1.29 ± 0.05	0.003	0.389	0.005
pH	7.412 ± 0.02	7.401 ± 0.02	7.396 ± 0.02	0.003	< 0.001	0.081
	**Time from centrifugation to analysis (N = 31)**			**P**		
	**0-10 min** **(Tube A)**	**30-40 min** **(Tube B)**	**90-100 min** **(Tube C)**	**Tube B *vs.* Tube A**	**Tube C *vs.* Tube A**	**Tube C *vs.* Tube B**
iCa, mmol/L	1.30 ± 0.04	1.29 ± 0.04	1.28 ± 0.06	0.021	0.027	0.717
pH	7.405 ± 0.02	7.407 ± 0.02	7.410 ± 0.02	0.695	0.024	0.282
Data presented as mean ± standard deviation. The difference was tested using the ANOVA analysis for repeated measures with Bonferroni post-hoc analysis. P < 0.05 was considered statistically significant. iCa - ionized calcium.						

**Table 3:** Results of regression analysis of iCa concentrations in arterial whole blood and venous serum, and time-dependent iCa concentrations in serum samples

**iCa, mmol/L**	**Slope (95% CI)**	**Intercept (95% CI)**	**Random differences,** **RSD ± 1.96 RSD interval**	**Cusum test for linearity**
**Arterial whole blood – analysed within 20 min, venous serum tubes centrifuged 30 min after sampling,** **analysed within 10 min**				
Arterial blood Serum	1.17 (0.91 to 1.50)	- 0.20 (- 0.60 to 0.11)	0.02 ± 0.03	P = 0.780
**Serum tubes centrifuged at different times after sampling (all tubes analysed 0-10 min after centrifugation)**				
15 min (Tube 1) 30 min (Tube 2)	1.00 (0.86 to 1.20)	0.01 (- 0.25 to 0.20)	0.01 ± 0.03	P = 0.450
15 min (Tube 1) 60 min (Tube 3)	1.00 (0.80 to 1.40)	- 0.01 (- 0.53 to 0.25)	0.02 ± 0.04	P = 0.860
30 min (Tube 2) 60 min (Tube 3)	1.00 (0.86 to 1.50)	- 0.02 (- 0.68 to 0.17)	0.02± 0.04	P = 0.620
**Serum tubes analysed at different times after centrifugation (all tubes centrifuged 30 min after sampling)**				
0-10 min (Tube A) 30-40 min (Tube B)	1.00 (0.83 to 1.50)	- 0.01 (- 0.67 to 1.20)	0.02 ± 0.04	P = 0.870
0-10 min (Tube A) 90-100 min (Tube C)	2.00 (1.29 to 5.00)*	- 1.34 (- 5.24 to -0.40)^†^	0.03 ± 0.06	P = 0.820
30-40 min (Tube B) 90-100 min (Tube C)	2.00 (1.00 to 3.50)	- 1.29 (- 3.25 to -0.01)^†^	0.04 ± 0.08	P = 0.610
*proportional difference. ^†^constant difference. iCa - ionized calcium. RSD - residual standard deviation. CI - confidence interval.				

**Figure 2:**
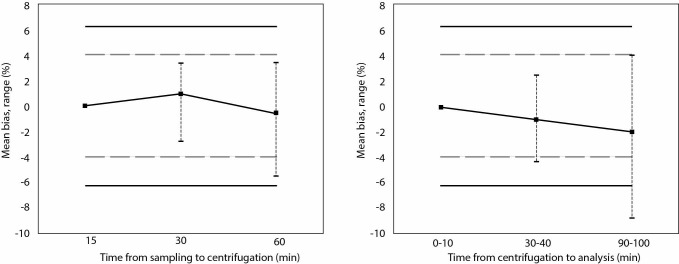
Time-dependent variations of iCa concentrations in serum samples from tubes that were centrifuged and analysed at different times. Dashed lines - analytically significant difference (4%). Solid lines - clinically significant difference (6.25%). iCa - ionized calcium.

Time-dependent variations of iCa concentrations in serum samples from tubes that were centrifuged and analysed at different times are presented in Figure 2. Calculated mean bias (%) did not exceed the analytically and clinically significant thresholds (CROQALM’s and RCV criteria), although bias (%) in some samples was higher than CROQALM’s and RCV criteria (Table 4). For three serum tubes that were centrifuged at different time after sampling, CROQALM’s criterion (4%) was exceeded in 2 of 30 subjects between Tube 3 and Tube 1 (60 *vs.* 15 min), and for the same subjects, the bias (%) between Tube 3 and Tube 2 (60 *vs.* 30 min) was higher than RCV (6.25%). For three serum tubes that were analysed at different times after centrifugation, CROQALM’s criterion was exceeded: in 1 of 31 subjects between Tube B and Tube A (30-40 *vs.* 0-10 min), in 11 subjects between Tube C and Tube A (90-100 *vs.* 0-10 min), in 12 subjects between Tube C and Tube B (90-100 *vs.* 30-40 min), while RCV was exceeded in 6 subjects between Tube C and Tube A, and between Tube C and Tube B.

**Table 4:** Comparison of the obtained bias with analytically and clinically significant differences for ionized calcium

		**Serum tubes centrifuged at different times after sampling**			**Serum tubes analysed at different times after centrifugation**		
	**Arterial blood *vs.* serum**	**15 min *vs.* 30 min**	**15 min *vs.* 60 min**	**30 min *vs.* 60 min**	**0-10 min *vs.* 30-40 min**	**0-10 min *vs.* 90-100 min**	**30-40 min *vs.* 90-100 min**
Mean bias ± SD (%)	0.22 ± 2.0	1.01 ± 1.45	- 0.50 ± 2.19	- 1.50 ± 2.53	- 0.97 ± 1.88	- 2.05 ± 4.11	- 1.09 ± 4.76
Range of bias (%)	- 3.39 to 4.51	- 2.27 to 3.42	- 5.56 to 3.44	- 8.97 to 3.15	- 4.31 to 2.56	- 9.11 to 4.10	- 10.53 to 6.06
Number of bias that exceed 4%*	1 of 25	0 of 30	2 of 30	2 of 30	1 of 31	11 of 31	12 of 31
Number of bias that exceed 6.25%^†^	0 of 25	0 of 30	0 of 30	2 of 30	0 of 31	6 of 31	6 of 31
*Analytically significant difference. ^†^Clinically significant difference.							

## Discussion

The CLSI recommendations suggest that the patient’s clinical status should influence the choice of sample type; whole blood with balanced heparin is the appropriate choice in acute and critical conditions requiring fast results, while anaerobically collected serum can be the best choice for routine diagnostic procedure (*7*). As previously mentioned, both type of specimen, whole blood and serum have their advantages, but also disadvantages that can affect iCa concentration. Our study showed no difference between iCa concentrations in arterial blood samples taken with balanced heparin analysed within 20 min after sampling and iCa concentrations in serum tubes with gel-separator centrifuged according to the manufacturer’s recommendations and analysed within 10 min after centrifugation (*14*). These results suggest that iCa results obtained from properly collected and timely analysed samples of arterial whole blood or venous serum can be used interchangeably.

It is interesting to note that current data on iCa differences in whole blood and serum are controversial; while one study presents higher iCa concentrations in serum samples, the other shows higher iCa concentrations in heparinized whole blood samples (*9*, *10*). The main reason for such ambiguous results lies in the fact that the studies were conducted under different conditions, including the types of heparin used, the time of analysis, the method of collecting samples and the analysis of arterial or venous whole blood that differ in pH, carbon dioxide and oxygen values (*17*). Actually, the opposite result of the aforementioned studies emphasizes the importance of pre-analytic factors in determining iCa concentration in whole blood and serum.

We also examined the effect of time between sampling and centrifugation and between centrifugation and analysis on iCa concentration in serum tubes that were left at room temperature. A longer period between sampling and centrifugation showed a statistically significant decrease in pH values in the serum sample, which can be explained by continued cellular metabolism during clotting, and lactate formation by glycolysis in erythrocytes and leukocytes (*5*). Calcium binding to albumin or other anions (phosphate, bicarbonate, sulphate, citrate, and lactate) is dependent on pH changes and decreases with a decrease of pH value, resulting in an increase of iCa concentration (*8*). In accordance with the aforementioned, the reason for higher iCa concentrations in tubes that were centrifuged 30 min after sampling (Tube 2) in relation to the tubes centrifuged 15 min after sampling (Tube 1) could lie in the pH decrease. However, it is interesting to observe that despite the decrease of pH in tubes centrifuged 60 min after sampling (Tube 3), iCa concentration was not higher, contrary, it was lower compared to iCa concentration in the tubes centrifuged 30 min after sampling. The reason for this result can only be speculated as a consequence of iCa binding to the newly created lactate. Therefore, bearing in mind results obtained by comparing iCa concentrations in arterial blood and venous serum, as well as the highest iCa concentrations in tubes centrifuged 30 min after sampling, it seems that the period between sampling and centrifugation recommended by the manufacturer of used test tubes is optimal for iCa determination in serum samples.

On the other hand, a longer time between centrifugation and sample analysis showed a statistically significant increase in pH values and a statistically significant decrease for iCa concentrations. It is well established that the sample exposed to room air loses carbon dioxide to the atmosphere and exhibits a pH rise which causes the consequent decrease of iCa (*6*). Although in our study the tubes were filled to the nominal volume and were not opened until the analysis was performed, pH increase and the decrease of iCa during 90 min may be attributed to the loss of carbon dioxide in the air space of the tubes. Also, it should be taken into account that some plastic materials absorb small amounts of carbon dioxide, leading to a gradual increase of pH (*5*). The same direction of change for iCa concentration and pH values was observed in the plasma samples from the lithium heparin tubes when the tubes were stored at room temperature for about 7 h, and in plasma samples exposed to air (*18*, *19*).

If only statistical significance is to be taken into consideration, one can conclude that serum tubes must be centrifuged 30 min after sampling and that iCa should be measured as soon as possible after sample centrifugation. On the other hand, if the mean bias is compared with the selected criteria for the analytically and clinically significant difference, it can be concluded that the observed changes are neither clinically nor analytically significant. However, since RCV is considered a relevant indicator of clinically significant difference because it takes into account the analytical variation of laboratory analysers and intra-individual biological variation, it is important to emphasize that RCV threshold was exceeded in some of the tested samples. A clinically significant difference was observed for two samples (of 30 examined samples) that were centrifuged 60 min after sampling and for six samples (of 31 examined samples) that were analysed 90 to 100 min after centrifugation. These results indicate the existence of inter-individual differences for iCa stability in the serum samples which can be explained by differences in pH changes, lactate formation, concentration of albumin and other anions. Highly variable effects on iCa concentration in serum samples as compared to whole blood, with a range of bias of - 0.09 to 0.05 mmol/L, were also observed in a study published by Toffaletti *et al.* (*9*). Unfortunately, this study did not provide data on the time of centrifugation and time of sample analysis.

To the best of our knowledge, our study for the first time represents time-dependent variability of iCa stability in serum samples. Since in the presence of acidosis or alkalosis the observed differences in iCa stability may be even more pronounced, the limitation of this study is that all samples tested had a pH value 7.4. Another limitation of the study is a small number of samples confirming the existence of inter-individual variations in iCa stability in serum samples and non-measurement of other components that may affect iCa stability.

In conclusion, properly collected and timely analysed samples of arterial whole blood and venous serum can be used interchangeably. Furthermore, our data imply that the time-dependent variation of iCa concentration in serum samples is individual. For most samples, iCa concentration will remain stable if the serum tube is centrifuged within 60 min after sampling and analysed within 90-100 min. However, in some samples, a clinically significant variation may be present. To avoid clinically significant variations, the serum tubes should be centrifuged within 30 min after sampling and the analysis should be performed within 30 min after centrifugation.
